# Characteristics of Transcriptional Activity in Nonlinear Dynamics of Genetic Regulatory Networks

**DOI:** 10.4137/grsb.s3119

**Published:** 2009-10-19

**Authors:** Simon Rosenfeld

**Affiliations:** National Cancer Institute, USA. Email: sr212a@nih.gov

**Keywords:** genetic regulatory network, transcription factors, microarrays, nonlinear dynamics, jacobian eigenvalues, asymptotic stability

## Abstract

Microarray measurements of mRNA abundances is a standard tool for evaluation of transcriptional activity in functional genomics. The methodology underlying these measurements assumes existence of a direct link between transcription levels, that is, gene-specific mRNA copy numbers present in the cell, and transcription rates, that is, the numbers of gene-specific mRNA molecules synthesized per unit of time. In this paper, the question of whether or not such a tight interdependence may exist is examined in the context of nonlinear dynamics of genetic regulatory networks. Using the equations of chemical kinetics, a model has been constructed that is capable of explicitly taking into consideration nonlinear interactions between the genes through the teamwork of transcription factors. Jacobian analysis of stability has shown that steady state equilibrium is impossible in such systems. However, phase space compressibility is found to be negative, thus suggesting that asymptotic stability may exist and assume either the form of limit cycle or of a chaotic attractor. It is argued that in rapidly fluctuating or chaotic systems, direct evaluation of transcription rates through transcription levels is highly problematic. It is also noted that even if a hypothetical steady state did exist, the knowledge of transcription levels alone would not be sufficient for the evaluation of transcription rates; an additional set of parameters, namely the mRNA decay rates, would be required. An overall conclusion of the work is that the measurements of mRNA abundances are not truly representative of the functionality of genes and structural fidelity of the genetic codes.

## 1. Introduction

DNA microarray measurements of mRNA abundances are widely recognized as a powerful tool for obtaining the whole-genome information regarding transcriptional activity. After the introduction of DNA microarrays^1^–^4^ and the initial rush of enthusiasm and great hopes,^5^–^7^ a period of sober evaluation of capabilities and pitfalls has emerged in which reproducibility has become a dominant concern.^8^,^9^ Thus, in the editorial preceding,^10^ the report summarizing a large-scale Microarray Quality Control Project (MAQC), this period of development is characterized as follows:

“... doubts linger about the reproducibility of microarray experiments at different sites, the comparability of results on different platforms and even the variability of microarray results in the same laboratory. After 15 years of research and development, broad consensus is still lacking concerning best practice not only for experimental design and sample preparation, but also for data acquisition, statistical analysis and interpretation. Though problematic for bench research, lack of resolution of these issues continues to even more seriously hamper translation of microarray technology into the regulatory and clinical settings ... Clearly, microarrays have a long way to go before they can be used to support regulatory decision-making or accurate and consistent prediction of patient outcomes in the clinic.”

Despite all these difficulties, there is a reasonable hope that relentless efforts towards improvement of technology, standardization of protocols and increasing cross-platform compatibility sooner or later will produce a desirable result, and the measurements will arrive at the status of satisfactory accuracy and self-consistency. However, it should be noted that many questions of interpretation of microarray measurements are still wide open and require much more analytical work. Among these questions is one of utmost importance: whether the measurements of mRNA abundances may serve at all as a basis for conclusions regarding transcriptional activity of corresponding genes.

Since the very inception of microarrays, these measurements have been termed *gene expression profiling*, and this term has been used interchangeably with *transcription profiling*. Such a terminology assumes that through the measurements of intracellular mRNA abundances (i.e. mRNA copy numbers) one can come to some conclusions regarding the status and/or activity of the corresponding genes. However, as is well known, each transcription event is supported by a team of transcription factors (TF), the proteins translated from a whole host of other mRNAs (e.g.^11^). This means that any gene-specific mRNA concentration is in a sense a product of the teamwork of cooperating genes, and in which the gene responsible for the mRNA in question may not even play a dominant role. In the literature, one may find numerous examples in which mRNA measurements are directly used for fitting the models of genetic regulatory networks.^12^–^19^ A good review of these efforts is given in.^20^ These examples clearly indicate that there is a widespread tendency to assume that measurements of mRNA abundances represent a reasonably solid basis for making the inferences regarding transcriptional activity of corresponding parent genes.

Is it really true that such a direct link between the mRNA concentrations within the cell and the transcription rates of corresponding genes does exist? In order to clarify this question, let us consider the following example. Suppose that a microarray experiment shows that a certain mRNA (say, mRNA-X) is under-expressed (that is, has a low copy number) in the control group (CG) as compared to that in the treatment group (TG). Would it necessarily mean that the Gene-X in the control group is *shut down* (or has very low activity) but in the treatment group it is active? A reasonable answer would be *maybe*. This is because along with a straightforward *yes* there is also a possibility that the genes in CG and TG are equally active, but due to some epigenetic reasons the mRNA-X in CG is chemically unstable and gets quickly dissolved in the cytoplasm leaving no traces of transcriptional activity. This example shows that mRNA abundance, apart from activity of the corresponding gene, depends on many other factors that may or may not be gene-specific. There is a constant influx of a large assortment of proteins from the cytoplasm to the nucleus serving as transcription factors (TF). Therefore, abilities of the individual genes to transcribe are also dependent on the availability of those factors, and fidelity of the gene’s genetic code may or may not play a role in maintaining a normal transcription rate. Even under the simplest assumption that ribosomes synthesize only one polypeptide chain from one mRNA molecule, a (large) number of other mRNAs are indirectly involved in the activity of each individual gene.

In more formal mathematical language, the question may be reformulated as follows. Suppose that *r_X_* is the concentration of mRNA-X molecules present in the cell, and *dr_X_*/*dt* = [*dr_X_*/*dt*]_+_ – [*dr_X_*/*dr*]_−_, where + and – signs distinguish transcription and degradation rates, respectively. The question we are trying to resolve is whether or not *r_X_* and [*dr_X_/dt*]_+_ are some-how linked to each other? A simple analogy may help to clarify the difference between *r_X_* and [*dr_X_/dt*]_+_. It is similar to that between someone’s income and his/her account balance. A frugal spender may have big savings (transcription level, *r_X_*) despite a modest income (transcription rate, [*dr_X_*/dt]_+_). An indiscriminate spender can squander his/her multimillion dollar income (transcription rate, [*dr_X_/dt*]_+_) and drive the estate to bankruptcy, that is, to a zero or a negative balance (transcription level, *r_X_*). Therefore, even complete knowledge of someone’s account balance generates no information regarding his/her income. In other words, there is no direct relation between *r_X_* and [*dr_X_/dt*]_+_. Such a relation may become known only after solving the dynamical equation for *r_X_*, that is *dr_X_/dt* = *F_X_* (*r_X_, r_Y_, r_X_*, …). However, due to numerous interactions in the system, the solution would depend on the states of all the interacting variables, therefore [*dr_X_/dt*]_+_ cannot depend on the *r_X_* only.

Attempts to formulate complex intracellular behavior using the concepts of nonlinear dynamics have been undertaken in many works.^21^–^24^ Essentially, this is the way in which the very concept of genetic regulatory network has come to existence. Up-to-date, experimental attempts to directly evaluate the differences and interdependencies between TRs and TLs have been scanty but all of them have produced remarkable results. Bernstein et al in experiments with *Escherichia coli* found no correlation between the transcript abundance and the transcript *stability* (in biology, this term is often used in lieu of the term mRNA *decay rate*).^25^ In the breakthrough time-course experiment,^26^ the TLs and TRs have been measured simultaneously in budding yeast. It was found that from a total of 5,500 TL-TR time-series pairs, about half turned out to be uncorrelated with each other. Cheadle et al come to the overall conclusion that “regulation of mRNA stability contributes significantly to the observed changes in gene expression,” and that “effective correlation of observed changes in gene expression with shared transcription regulatory elements remain difficult to demonstrate convincingly”.^27^,^28^ The question of correlation between transcription levels and transcription rates in a multidimensional system has been addressed in the work by this author.^29^ It was shown by simulation that in a network of very high complexity (i.e., link density), the dissimilarities between temporal behaviors of TL and TR increase as the network dimension increases because of a longer chain of intermediate events leading to each act of gene expression. These considerations show that the gene’s TR is not something that is easy to infer from the measurements of TL. Apart from those indicated above, there are a number of other reasons that make it problematic to directly evaluate transcriptional regulation from microarray experiments. Detailed discussion of these additional aspects of the problem goes beyond the scope of this paper; a good review is available from.^30^

The goal of this paper is to discuss the prerequisites for the very possibility that certain TL-TR relationships could exist. An overall message we intend to convey is that DNA microarray data cannot serve as an unambiguous basis for making judgments regarding the ability of genes to generate transcripts.

## 2. Mathematical Descriptions of Gene Expression Biochemistry

Mathematical description of biochemical reactions is deeply rooted in statistical thermodynamics, and its special branch called chemical kinetics. At its very core, a system of chemical reactions may be written in the form of the *Law of Mass Action*

(1.1) $$\begin{array}{ll} \frac{dx_{i}}{dt} & =F_{i}(x_{1},\ldots ,x_{2N})\\ & =\phi_{i}\prod_{m=1}^{2N}x_{m}^{P_{im}}-\psi_{i}\prod_{m=1}^{2N}x_{m}^{Q_{im}},i=1,\ldots ,2N \end{array}$$

where *N* is the number of protein types present in the system which is considered to be identical with the number of mRNA types; *ϕ_i_*, *ψ_i_* are the 2*N*-vectors of kinetic rates, and *P_im_, Q_im_* are the 2*N* × 2*N* matrices of stoichiometric coefficients in the direct and inverse reactions, respectively.^31^,^32^ Depending on the nature and complexity of the system under investigation, the quantities {*x_i_*} may represent concentrations of various biochemical constituents participating in the process, including individual molecules or their aggregates. The system (1.1) is also known as S-System. Applicability of S-Systems to description of biochemical processes and to fitting microarray data have been extensively discussed in the literature.^23^,^31^–^36^

In order to adapt a general system (1.1) to the specific goals of describing the biochemistry of gene expression, we split it into two parts. We introduce the N-vectors of the protein copy numbers, *p*, and of the mRNA copy numbers, *r* (per cell). Equation (1.1) is now rewritten as follows

(1.2) $$\begin{array}{ll} \frac{dp_{i}}{dt}= & \alpha_{i}\prod_{k=N+1}^{2N}r_{k}^{P_{i\;k}}\prod_{k=1}^{N}p_{k}^{P_{i\;k}}-\beta_{i}\prod_{k=N+1}^{2N}r_{k}^{Q_{i\;k}}\prod_{k=1}^{N}p_{k}^{Q_{i\;k}},\\ & i=1,\ldots ,N\\ \frac{dr_{i}}{dt}= & \gamma_{i}\prod_{k=1}^{N}p_{k}^{P_{i\;k}}\prod_{k=N+1}^{2N}r_{k}^{P_{i\;k}}-\delta_{i}\prod_{k=1}^{N}p_{k}^{Q_{i\;k}}\prod_{k=N+1}^{2N}r_{k}^{Q_{i\;k}},\\ & i=N+1,\ldots ,2N \end{array}$$

where, in accordance with notation of (1.1),

(1.3) $$\begin{array}{l} \alpha_{1:N}=\phi_{1:N},\beta_{1:N}=\psi_{1:N},\\ \gamma_{1:N}=\phi_{\left(N+1):(2N\right)},\delta_{1:N}=\psi_{\left(N+1):(2N\right)} \end{array}$$

Equations (1.2) depict gene expression as a closed circulatory system in which the synthesis of mRNA molecules (transcription) is followed by the synthesis of proteins by ribosomes (translation) and return of the proteins back to the regulatory sites as transcription factors for other genes. This is, of course, a highly idealized schema in which many secondary processes are ignored. However, such an idealization is suitable for our purposes. We intend to demonstrate that even in such a restrictive system the relations between transcription levels and transcription rates may be very complex and fuzzy. In a real life situation, when a number of secondary factors distort even this restrictive functionality, these relationships may be even fuzzier or absent altogether.

The first term in the first Equation (1.2), $A_{i}=\alpha_{i}\prod_{k=N+1}^{2N}r_{k}^{P_{i\;k}}\prod_{k=1}^{N}p_{k}^{P_{ik}},$ describes production of proteins by ribosomes. Coefficient *A_i_* here is the *rate of translation*, i.e. the number of proteins of *i*-th type synthesized by ribosome per unit of time. According to the Central Dogma in molecular biology, only one mRNA type participates in the translation of the *i*-th protein, and this is the *i*-th mRNA. Also, with a good approximation, one may surmise that other proteins are not involved in the translation of the *i*-th protein. This means that *A_i_* = *α_i_r_i_*.

The second term in the first Equation (1.2), $B_{i}=\beta_{i}\prod_{k=N+1}^{2N}r_{k}^{Q_{i\;k}}\prod_{k=1}^{N}p_{k}^{Q_{i\;k}}$, describes disappearance of the *i*-th protein. In our idealized system, such a disappearance occurs only when the protein reaches the designated regulatory site, associates with DNA and becomes a TF. According to the Central Dogma, mRNAs do not participate in this process. We also take into consideration that there are no chemical reactions between the proteins within the regulatory site which could produce new proteins. All this means that *B_i_* = *β_i_p_i_*.

The first term in the second Equation (1.2), $C_{i}=\gamma_{i}\prod_{k=1}^{N}p_{k}^{P_{i\;k}}\prod_{k=N+1}^{2N}r_{k}^{P_{i\;k}}$, describes *transcription*, that is the synthesis of the mRNA of *i*-th type. Other mRNAs do not participate in this process. As is well known, transcription requires participation of a large number of proteins, typically from 30 to 100, serving as TFs and as building blocks for the RNA Polymerase, the centerpiece of decoding machinery.^11^ Therefore, a general form of this term is $C_{i}=\alpha_{i}\prod_{k=1}^{N}p_{k}^{P_{i\;k}}$. Collections of the proteins serving as TFs are generally gene-specific (which, of course, does not preclude some overlap with other genes). Typically, the stoichiometric matrices, *P_ik_*, are sparse with the majority of elements being zero and the rest of them being small integers. The number of transcription factors, $\nu_{i}=\sum_{k=1}^{N}P_{ik}$ per gene is a characteristic of the complexity of the system; in,^29^ an average of these quantities $\lambda =\sum_{i=1}^{N}\nu_{i}/N$ has been termed the *index of complexity*. In general network theory, the parameter, ζ = λ*N*, is also called *link density*.

The second term in the second Equation (1.2), $D_{i}=\delta_{i}\prod_{k=1}^{N}p_{k}^{Q_{i\;k}}\prod_{k=N+1}^{2N}r_{k}^{Q_{i\;k}}$, describes decay of mRNAs in the process of templating by ribosome and subsequent dissolution in the cytoplasmic environment. Since after translation the mRNA molecules do not return back to transcription machinery, the decay rate may be written simply as *D_i_* = *δ_i_r_i_*.

Summarizing all these considerations (and slightly simplifying indexing), we rewrite (1.2) as

(1.4) $$\frac{dp_{i}}{dt}=\alpha_{i}r_{i}-\beta_{i}p_{i},\frac{dr_{i}}{dt}=\gamma_{i}\prod_{k=1}^{N}p_{k}^{P_{ik}}-\delta_{i}r_{i},i=1,\ldots ,N$$

Similar equations (though without a disclosure of the analytical structure of the mRNA production term) for describing gene expression kinetics has been proposed in.^21^ In the work^29^ by this author, a slightly more general form of equation (1.4) has been used for studying stochastic oscillation in multidimensional regulatory networks.

In the considerations leading to (1.4), the author does not claim any novelty; they are provided solely for keeping the paper self-contained and for highlighting the biological origins of assumptions. Equations (1.4) bear much similarity to those proposed in.^20^,^37^ The difference is that in the latter works the elements of stoichiometric matrices are allowed to be any real numbers, both positive and negative, thus providing a wider flexibility in model fitting. However, such an algorithmic advantage comes at the cost of losing direct link to the equations of chemical kinetics in which the elements of stoichiometric matrices are nothing else as the numbers of transcription factors; these are the positive integers. In addition, as will be shown later in this paper, the non-negativity of stoichiometric matrices is an important prerequisite in the network analysis, in general, and in the analysis of dynamical stability, in particular.^38^

Despite a fairly innocuous look, equations (1.4) are, in fact, very complex, and there is no easy way to deal with them. The difficulty is residing in the mRNA production term, *C_i_*, which is a polynomial function of very high order in the protein concentrations. The way this problem was dealt with in^21^ consisted of linearization around some hypothesized steady state. In,^29^ the existence of such a steady state has been questioned, and it was shown that without special, very stringent conditions imposed on the coefficients in (1.4) such a steady state could not exist. Hence, instead of linearization, the nonlinear term has been replaced by a random force thus converting the system to a linear stochastic differential equation. Stochastic properties of the random force have been derived iteratively through a fairly involved successive approximation procedure.

As stated above, the goal of this paper is to explore whether or not measurement of the transcription levels, *r_i_*, may be used for inferring information regarding the transcription rates *C_i_*. For the reasons to be explained later in this paper, a specific form of dependence of transcription rates on protein concentrations, that is *C_i_* = *C_i_* (**p**) is not of crucial importance in this consideration; the only prerequisite is just recognition of an evident fact that there are many TFs facilitating each transcription, and that these TFs are predominantly the protein molecules. Four questions should be considered in this context. First, if an equilibrium state in the system described by (1.4) did exist, then would it be possible to express the transcription rates through the transcription levels? Second, does an equilibrium state indeed exist in the system described by (1.4)? Third, if an equilibrium state does exist then can it be stable? Fourth, if an equilibrium state does exist but it is not stable then what kind of dynamical behavior could be reasonably expected of such a system?

Prior to exploring these issues, some conventions regarding vocabulary are introduced.

## 3. Some Terminology

Since in different contexts the words stability, equilibrium, steady state and such are often used quite differently, there is a need to introduce the meanings of these words as they are used in this paper.

*Fixed point* is a point in the space of all the system’s variables, i.e. protein and mRNA concentrations (a.k.a. *phase space*) in which the time derivatives are zero, i.e. **ṗ**(*t*) = {*dp_i_/dt*} ≡ 0 and **ṙ**(*t*) ≡{*dr_i_/dt*}≡ 0. By this definition, the fixed point does not move in phase space with time. The fixed point is also called the *equilibrium point*.

Equilibrium at a fixed point may be stable or unstable. *Stable equilibrium* is a state for which small deviations from the fixed point generate internal forces that push the system back towards equilibrium. These forces may occur due to the existence of *negative feedback loops. Unstable equilibrium* is a state for which small deviations from the fixed point generate internal forces that push the system even further from equilibrium, often causing the system’s collapse. These forces may indicate existence of the *positive feedback loops*.

The term *steady state* may refer to both motionless position of the system at the stable fixed point and stationary oscillations in the vicinity of this position. In the latter case, the term *steady state* may be used interchangeably with the term *limit cycle*. Experimentally, such a motion is often interpreted as a *stationary stochastic process*, that is, such a process in which statistical characteristics do not change with time.

*Asymptotic dynamic stability* is the property of a dynamical system to approach, with time, a certain domain in phase space and stay within this domain forever. This domain is often called the *attractor*. Motions within this domain may include *stable oscillations or chaotic orbits*.

The concept of dynamical stability is fundamentally different from the concept of biological robustness. An in-depth discussion of this difference has been given by this author in a number of previous works.^39^–^42^ See the Discussion section for some additional details and references.

## 4. Interrelations Between Transcription Levels and Transcription Rates at Equilibrium

The fixed point, $\left\{r_{i}^{0},p_{i}^{0}\right\}$, of the system (1.4) is the solution of simultaneous algebraic equations

(1.5) $$\alpha_{i}r_{i}^{0}=\beta_{i}p_{i}^{0},C_{i}({\text{p}}^{0})=\delta_{i}r_{i}^{0},i=1,\ldots ,N$$

**Figure 1.**
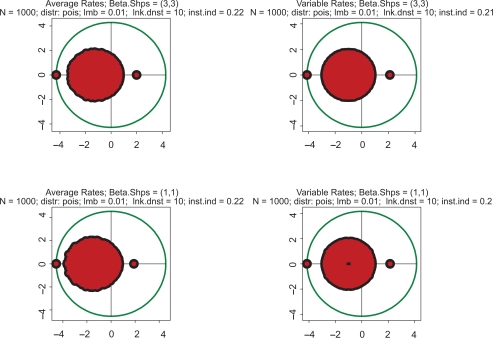
Typical spectra of eigenvalues. Top row: kinetic rates are distributed uniformly (beta with shapes 1 and 1). Bottom row: distribution is unimodal (beta shapes 3 and 3. Left column: rates are constant and equal to the averages of those in right column.

According to a number of sources,^36^ including the works by this author,^43^,^44^ the solution to (1.5) does exist and is unique; the exact analytical form of the solution will be addressed later. It is important to realize though that even if a fixed point does exist, the determination of the transcription rate is only possible if the kinetic rates of degradation, {*δ_i_*}, are known. This fact has recently transpired in a number of publications (e.g.^45^,^46^). As observed in a number of experimental studies, the value of parameters {*δ_i_*} may vary within orders of magnitude. Thus, it has been shown in^47^ that in mouse embryonic stem cells, the mRNA half-lives are distributed approximately lognormally (see Fig. 1F in^47^) with the mode at ∼5.5 hours and range from 2 to 24 hours (measured) and beyond (not measured). The study of time-course kinetics and half-lives of ∼1500 mRNAs in microbial cells has been undertaken in.^48^ The half-lives have been found to vary from less than 30 sec to more than 20 min. An important observation made in this work is that the half-lives are clustered into comparatively tight groups, and that observed co-regulation of genes may in fact originate from the mere fact that the half-lives of corresponding mRNAs are close to each other. It is not yet known what biochemical factors influence degradation rates, {*δ_i_*}. Preliminary considerations, mixed with intuition, suggest that the mRNA decay rates are not sequence-specific; rather they are dependent on the mRNA’s length and some structural properties such as the number of exon junctions per open reading frame.^47^ Authors^48^ even go as far as to claim: “Based on our present knowledge, it seems clear that determinants of mRNA stability in bacteria cannot be identified from the primary sequence of mRNA.” Obviously, all this means that the mRNA decay rates are not gene-specific, and therefore observed mRNA abundances have little to do with the transcription rates of the genes they were transcribed from. This conclusion is of crucial importance for the interpretation of microarray data. Since {*δ_i_*} are highly variable and *unknown*, the { *C_i_* (**p**^0^)} cannot be evaluated through the $\left\{r_{i}^{0}\right\}$ even if the latter are experimentally determined and available. Standard microarray protocols are capable to detect only differences in mRNA abundances and do not include concurrent measurements of mRNA half-lives. High mRNA abundances may simply originate from their long half-lives which, as shown in,^47^,^48^ are not gene-specific. All this means that, unless the mRNA abundances and mRNA decay rates are measured simultaneously in the same experimental settings, the transcription rates cannot be appropriately identified *even in the state of equilibrium*.

## 5. Jacobian (in)Stability of the Equilibrium

Let us suppose now, quite hypothetically, that the mRNA decay rates, {*δ_i_*}, somehow became available. Would it be sufficient for estimating the transcription levels from transcription rates? To this end, the question of stability at equilibrium comes into focus. This is because it is utterly unlikely that if the equilibrium is unstable then the system would reside in this state for a long time. It would be similar to a pyramid being carefully balanced on its tip and stably residing in this position for a long time despite all kinds of disturbances around. It is illustrative that for the equilibrium of a pyramid the forces should be balanced in two dimensions (two angles of rotation), whereas in a genetic regulatory system the balancing should be applied to the dimensions in hundreds and thousands. If a system departs from the fixed point, then the only link between transcription level and transcription rate expressed by the second equation in (1.5) disappears, and the very question of relations between them becomes immaterial.

A standard way of analyzing stability consists in linearization of dynamics in the vicinity of the fixed point, obtaining the Jacobian matrix and computing the spectrum of its eigenvalues. Presence in the spectrum of eigenvalues with positive real parts is an indicator of instability.^49^ A detailed derivation of the Jacobian matrix, *J_im_*, for the systems described by (1.1) is given in Ref^43^ by this author. The Jacobian matrix is

(1.6) $$J_{im}=\frac{1}{\tau_{i}}(P_{im}-Q_{im});\;\;\;i,m=1:(2N)$$

where

(1.7) $$\tau_{i}=\phi_{i}\text{exp}\left\{\sum_{m=1}^{N}W_{im}\text{ln}\left(\frac{\phi_{m}}{\psi_{m}}\right)\right\},\text{W}=(\text{P}-\text{I})\;{(\text{P}-\text{Q})}^{-1}$$

Symbolically, the specifications of stoichiometric matrices for gene expression kinetics are as follows

(1.8) $$\text{P}=\left\|\begin{array}{ll} {\text{P}}_{\text{prot}}^{\text{prot}} & {\text{P}}_{\text{prot}}^{\text{mRNA}}\\ {\text{P}}_{\text{mRNA}}^{\text{prot}} & {\text{P}}_{\text{mRNA}}^{\text{mRNA}} \end{array}\right\|;\text{Q}=\left\|\begin{array}{ll} {\text{Q}}_{\text{prot}}^{\text{prot}} & {\text{Q}}_{\text{prot}}^{\text{mRNA}}\\ {\text{Q}}_{\text{mRNA}}^{\text{prot}} & {\text{Q}}_{\text{mRNA}}^{\text{mRNA}} \end{array}\right\|$$

where

(1.9) $$\begin{array}{l} {\text{P}}_{\text{prot}}^{\text{prot}}={\text{P}}_{\text{mRNA}}^{\text{mRNA}}=\left\|0\right\|,\\ {\text{P}}_{\text{prot}}^{\text{mRNA}}=\text{diag}(1,\ldots ,1)=\text{I},\\ {\text{P}}_{\text{mRNA}}^{\text{prot}}=\left\|\varepsilon_{ij}\right\|,\\ {\text{Q}}_{\text{prot}}^{\text{prot}}={\text{Q}}_{\text{mRNA}}^{\text{mRNA}}=\text{I},\\ {\text{Q}}_{\text{prot}}^{\text{mRNA}}={\text{Q}}_{\text{mRNA}}^{\text{prot}}=\left\|0\right\| \end{array}$$

The only matrix requiring some explanation is ${\text{P}}_{\text{mRNA}}^{\text{prot}}$ describing stoichiometry of transcription. Its components, *ɛ_ij_*, indicate how many proteins of type *j* serve as a TF for the gene *i*. Since only a comparatively small fraction of all the protein species comprising the proteome participate in each transcription, the majority of all *ɛ_ij_* are zeros, the majority of non-zeros are ones, and sometimes *ɛ_ij_* may be equal to two. Should more specific data become available, these assumptions may be easily modified. A convenient way to express this property mathematically is to assume that *ɛ_ij_* are randomly drawn from the Poisson or binomial distribution. This is not supposed to mean that the stoichiometric coefficients are indeed random; it is just a technical way of saying that the parameterizations of stoichiometry and kinetics are entirely independent.

We introduce two auxiliary matrices

(1.10) $$\text{U}=\text{diag}(\tau_{1}^{-1},\ldots ,\tau_{N}^{-1}),\text{V}=\text{diag}(\tau_{N+1}^{-1},\ldots ,\tau_{2N}^{-1})$$

According to eq. (1.6) and (1.8), the Jacobian matrix is representable as

(1.11) $$\text{J}(0)=\left\|\begin{array}{cc} \text{A} & \text{B}\\ \text{C} & \text{D} \end{array}\right\|,$$

where **A** = −**U**, **D** = −**V**, **B** = **U**, **C** = **Vɛ**. We need to find the solutions of the characteristic equation

(1.12) $$\text{J}(\lambda )=\left\|\begin{array}{cc} \text{A}-\lambda \text{I} & \text{B}\\ \text{C} & \text{D}-\lambda \text{I} \end{array}\right\|=0$$

Applying Shur’s formula for the determinants of block matrices,^50^ we reduce **J** (λ) to

(1.13) det J(λ)=det[VUε−(V+λI)(U+λI)]

In order to envision a general structure of the spectrum (1.13), we first make some inessential simplification. After that, we demonstrate numerically that this simplification is quite satisfactory and does not distort the results. To this end, we replace *diag* (**U**) and *diag* (**V**) by their mean values, which, according to eq. (10) in^43^, are equal to 1, thus bringing (1.3) to

(1.14) $$\text{det}\;\text{J}(\lambda )=-\text{det}\left\|\varepsilon -{\left(1+\lambda \right)}^{2}\text{I}\right\|$$

Denoting Λ = (1 + λ)^2^, we arrive to the characteristic equation

(1.15) det‖ε−ΛI‖=0

If Λ_1:_*_N_* are the eigenvalues of ||**ɛ**||, then the roots of **J**(λ) = 0 are $\lambda_{1:(2N)}=\pm \Lambda_{1:N}^{1/2}-1$. The problem is now reduced to finding the spectrum of ||**ɛ**||. Since ||**ɛ**|| is non-negative, then, according to the fundamental Perron-Frobenius theorem,^50^ there always exists a unique real positive eigenvalue, Λ_max_, which is greater than the moduli of the rest of the eigenvalues. In order to demonstrate instability, it is sufficient to show that Λ_max_ >1. In this case, the λ—spectrum contains at least one eigenvalue with a positive real part, λ_max_ > 0.

We have performed an extensive series of numeric computations of Jacobian spectra under wide ranging assumptions regarding kinetic rates and stoichiometric matrices. Several important findings have resulted from these computations. First, it turns out that not only λ_max_ > 0, but also there exists a large set of other eigenvalues with positive real parts. We will refer to this property as *massive instability*. Second, the eigenvalue spectra are fairly robust with respect to the replacement of actual *diag* (**U**) and *diag* (**V**) by their means. Therefore, the simplification which led to (1.14) (that is, the replacement of *diag* (**U**) and *diag* (**V**) by their means) is justified. Third, such massive instability takes place under widely varying assumptions regarding distributions of kinetic rates and stoichiometric coefficients. To the best of the author’s knowledge, these results are not yet known and may represent some interest on their own, beyond the context of this paper.

Examples supporting the above three statements are given in the following Figures. In each of them, Jacobian matrices of order 1000 have been analyzed. Figure 1 shows the results of computations for two drastically different distributions of kinetic rates. In the top row, kinetic rates are distributed uniformly (beta distribution with shapes 1 and 1). In the bottom row, the distribution is unimodal (beta with shapes 3 and 3). Comparison between rows shows that the distributional shapes of kinetic rates are not of much importance for overall structure of the spectrum, and therefore have a little impact on its stability. The graphs in the right column depict the spectra obtained with variable kinetic rates, whereas in the left column are shown those with the constant rates. It is seen that they are identical. Importantly, for all four cases the fractions of all roots located in the right half of complex plane (called hereafter the *instability index*) are identical. This is an illustration of the fact that replacement of the kinetic rates by their averages has little or no impact on the spectrum; this means that the assumption underlying the derivation of (1.14) is well justified. (For the reader’s convenience, the densities of the beta distribution for various combinations of shape parameters are given in Fig. 8).

**Figure 2.**
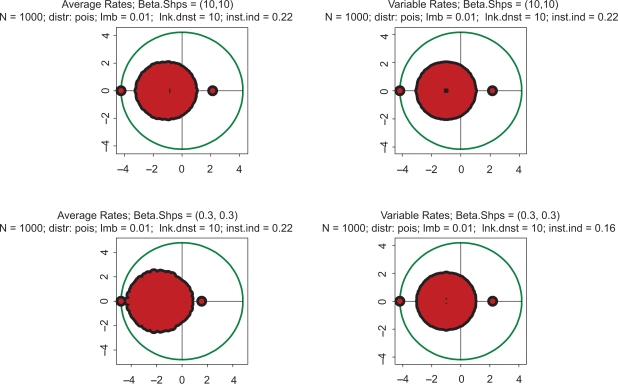
Spectra of eigenvalues for drastically different distributions of kinetic rates. Top row: kinetic rates are unimodal with very little variations (beta with shapes 10 and 10. Bottom row: distribution is bimodal with sharp peaks at zero and one (beta with shapes 0.3 and 0.3. Left column: rates are constant and equal to the averages of those in right column.

These properties largely remain intact in even more extreme cases. Eigenvalue spectra for drastically different distributions of kinetic rates are depicted in Figure 2. In the top row, the kinetic rates are unimodal with very little variation and a sharp peak in the center of the interval (beta with shapes 10 and 10). This setting represents a highly hypothetical case of almost constant kinetic rates across the entire spectrum of biochemical reactions. In the bottom row, the distribution is bimodal with sharp peaks at the edges (beta with shapes 0.3 and 0.3). This is the case representing a mixture of very fast and very slow processes. Again, in the left column the rates are constant, and in the right column variable. Comparison reveals some differences, but neither violate the key conclusion that all the spectra are unstable, nor do they lead to drastically different instability indexes.

**Figure 3.**
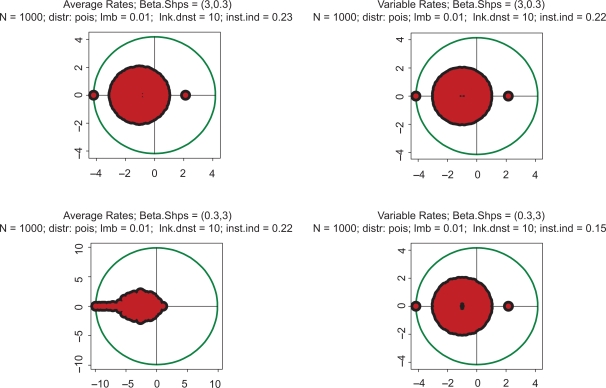
Spectra of eigenvalues for drastically different distributions of kinetic rates. Top row: kinetic rates are unimodal with sharp peak at the right end of the interval (beta with shapes 3 and 0.3). Bottom row: kinetic rates are unimodal with sharp peak at the left end of interval (beta with shapes 0.3 and 3). Left column: rates are constant and equal to the averages of those in right column.

The same pattern is seen in Figure 3. In the top row, the kinetic rates have steep elevation to the right end of the interval (beta with shapes 3 and 0.3). Such a distribution of kinetic rates depicts the dominance of fast processes. In the bottom row, the kinetic rates have steep elevation to the left end of the interval (beta with shapes 0.3 and 3), which corresponds to the dominance of slow processes. There are some differences in the spectra, but all of them are unstable with approximately identical instability indexes.

**Figure 4.**
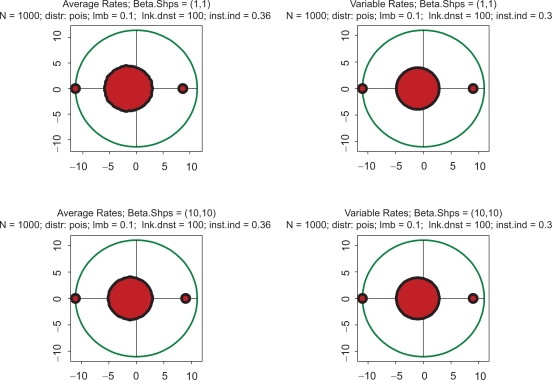
Top row: kinetic rates are distributed uniformly (beta with shapes 1 and 1). Bottom row: distribution is unimodal (beta shapes 3 and 3. Left column: rates are constant and equal to the averages of those in right column. The difference with Figure 1 is that the link density here is 100.

**Figure 5.**
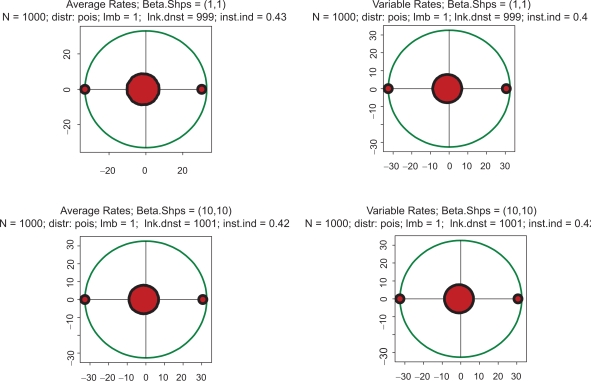
Top row: kinetic rates are distributed uniformly (beta with shapes 1 and 1). Bottom row: distribution is unimodal (beta shapes 3 and 3. Left column: rates are constant and equal to the averages of those in right column. The difference with Figure 1 is that the link density here is 1000.

All the above computations have been made with the Poisson parameter 0.01. With a genome size of 1000, this means that the link density (i.e. average number of protein serving as transcription factors per gene) is about 10. Figure 4 shows the impact of increasing complexity on the eigenspectrum. The kinetic rates in this Figure are the same as in Figure 1, but there is a difference in stoichiometry: the link density is ten times greater. Again, all four spectra are almost indistinguishable, but the instability index is drastically higher than in Figures 1–3. The conclusion which may be derived from this example is that *instability increases with increasing complexity*. Just out of curiosity, we have also looked into an utterly unrealistic case when the link density approaches the dimension of the system. In the gene expression context, it would mean that an overwhelming majority of the protein species serve as transcription factors in each particular gene. This case is depicted in Figure 5. Instability has increased even further, and the spectrum became almost symmetric.

The general structure of the spectra in Figures 1–5 may be understood from the following considerations. Matrix ||**ɛ**|| can be rescaled to the form ||**ɛ**|| = ζ ||**Ξ**|| where ζ is the *link density* (introduced in the Section 2) and ||**Ξ**|| is a *stochastic matrix*, that is, the one in which all the within-row sums are equal to 1. As known from the matrix theory (e.g.^50^ XIII.6), the largest eigenvalue of a stochastic matrix is 1. Therefore, the largest eigenvalue of the Jacobian, **J** (0), is simply

(1.16) $$\lambda_{\text{max}}=\zeta^{½}-1$$

**Figure 6.**
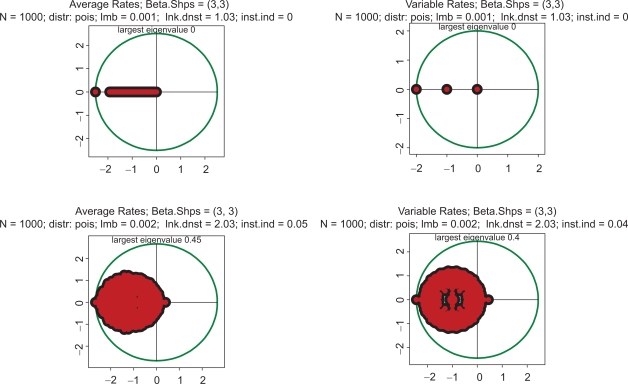
All four pictures: distribution is unimodal (beta shapes 3 and 3. Top row: link density is 1. Bottom row: link density is 1. Left column: rates are constant and equal to the averages of those in right column.

Indeed, this relation precisely holds in all the cases presented in Figures 1–5. Formally speaking, according to (1.16), the system is unstable for any ζ > 1. In the theoretical limit, ζ → 1, which would mean that on average there is only one protein per gene serving as a TF, the system becomes linear, the largest eigenvalue tends to zero, and the spectrum shows only pure attenuations without oscillations (Fig. 6, top row). However, if the link density increases to 2 (two proteins per regulatory site) then even this comparatively weak nonlinearity immediately moves the system to instability, the largest egenvalue becomes ∼2^½^ 1 − 0.41, and the spectrum manifests oscillatory motions with a large assortment of frequencies, attenuations and excitations (50 eigenvalues) (Fig. 6, bottom row). It is obvious that with link densities from 30 to 100,^11^ typical for gene expression, any regulatory dynamics is located deeply within the domain of strong instabilities.

## 6. Limitations and Generalizations

The conclusion made in the previous Section may have far reaching implications for understanding the dynamics of genetic regulation. Therefore, a natural question arises whether or not a restrictive model (1.4) used for the above analysis of stability indeed warrants such a strong conjecture. In this context, several considerations are in order. First, the specific form (1.4) of a biochemical system can be easily modified to accommodate other processes. For example, one may surmise that mRNA degradation is assisted by other proteins; in this case the ${\text{Q}}_{\text{prot}}^{\text{mRNA}}$ is non-zero. It is also conceivable that transcription may be assisted by some mRNAs. In this case, the matrix ${\text{P}}_{\text{mRNA}}^{\text{mRNA}}$ should be populated by non-zero stoichiometric coefficients. Other modifications and adjustments are also possible. Each such augmentation moves the system towards greater complexity, and therefore toward greater nonlinearity and instability. Although of some interest, there is no need to perform stability analysis for each such modification individually. In the works^43^,^44^ by this author, stability has been studied for the system in its general form (1.1). In the author’s Ref,^29^ the Generalized Law of Mass Action has been used instead of a simpler form (1.2). All these cases show various degrees of instability per minimal assumptions regarding interactions. Actually, the only significant assumption that would be sufficient for instability is that the interactions of some sort do exist.

**Figure 7.**
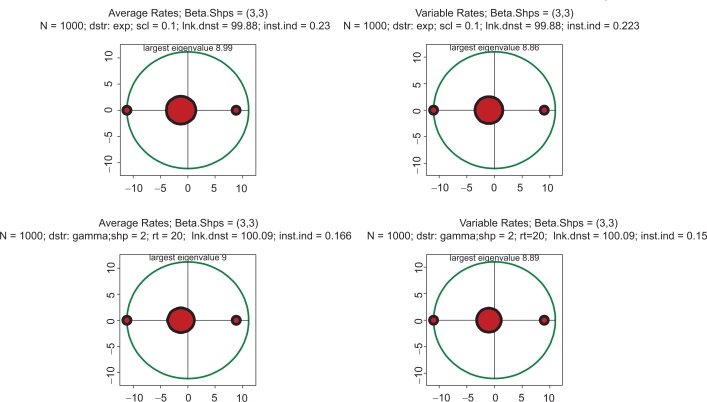
All four pictures: distribution of kinetic rates is unimodal (beta shapes 3 and 3), and link density is 100. Top row: stoichiometric coefficients are distributed exponentially with scale = 0.1. Bottom row: stoichiometric coefficients are distributed as gamma with shape = 2 and rate = 20. Both distributions produce the same mean = 0.1. Left column: kinetic rates are constant and equal to the averages of those in right column.

**Figure 8.**
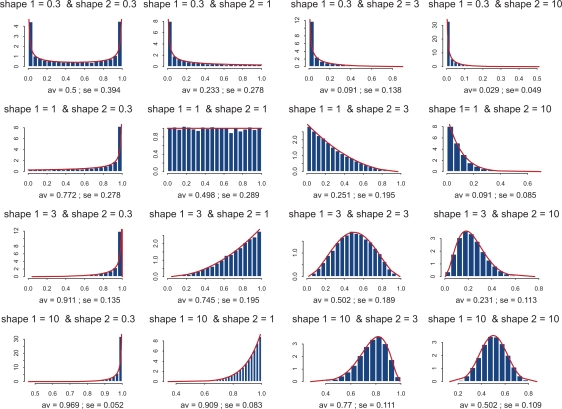
Histograms of the beta distributions for various combinations of shape parameters.

The very applicability of the concepts of chemical kinetics to intracellular biochemistry may also be questioned. To this end, the concepts of stoichiometry borrowed from low-dimensional chemistry between small (in the biochemical sense) molecules may not be quite adequate. To address this concern, simulations have been performed in which stoichiometric matrices were replaced by more general forms rather than simply containing only small positive integers. All the results regarding instability remain generally valid in these cases too. Thus, Figure 7 gives an example of spectra with exponentially (top row) and gamma (bottom row) distributed stoichiometric coefficients. It is quite remarkable that, despite such big differences of the distributional shapes, the portraits of stability are literally identical to those obtained with the Poisson distributed stoichiometry. This result allows one to hypothesize that perhaps stoichiometry may be expressed in any functional form provided that the stoichiometric coefficients are positive; link density is the only parameter which seems to be of significance. Stated differently, it may be said that stability is mostly an epiphenomenon of the network’s topology and fairly independent of its kinetics. Similar conclusions have been made in a number of other sources, e.g.^51^,^52^ although using quite different theoretical approaches.

Moving to the next level of abstraction, one should recall the result by Tournier^53^ saying that in the vicinity of fixed point, any nonlinear system may be represented through the S-system.^31^,^36^ In the Ref^44^ by this author, this general statement has been specified for a class of nonlinear systems which can be called *competitive: d***x**/*dt* = **F(Px)** – **G(Qx)**. In the vicinity of the fixed point this system is representable as

(1.17) $$\begin{array}{ll} \frac{dx_{i}}{dt} & =\Phi_{i}(t\;|\;{\text{x}}_{0})\\ & =\alpha_{i}\text{exp}\left[\sum_{k}\xi_{i}P_{ik}x_{k}\right]-\beta_{i}\text{exp}\left[\sum_{k}\eta_{i}Q_{ik}x_{k}\right] \end{array}$$

with

$$\begin{array}{l} \xi =\nabla_{\text{U}}\text{F};\eta =\nabla_{\text{V}}\text{G};\alpha =\text{exp}[\text{F}({\text{U}}^{0})-\xi {\text{U}}^{0}];\\ \beta =\text{exp}[\text{G}({\text{V}}^{0})-\eta {\text{V}}^{0}],{\text{U}}^{0}=\text{P}{\text{x}}^{0};{\text{V}}^{0}=\text{Q}{\text{x}}^{0} \end{array}$$

In a sense, this means that in a competitive system, a large-scale dynamics may be considered, at least locally, as a *kind of chemistry* per appropriate definitions of chemical constituents and kinetic rates. This also means that the above results regarding stability may make some sense in a much wider class of nonlinear dynamical systems. In particular, the Jacobian matrix of (1.17) is generally neither symmetric nor anti-symmetric; hence, its eigenvalues are complex numbers with both negative and positive real parts, what would indicate dynamic instability.

A major limitation of ordinary differential equations, such as (1.1), in application to intracellular biochemistry is that they are valid only for the *well stirred* systems. In such systems, any chemical constituent produced anywhere in the system is assumed to become immediately available for all the chemical processes throughout the system. To some extent, this assumption is acceptable in prokaryotic cells where the tightly coiled DNA molecule is surrounded by ribosomes in a somewhat random manner and located in close proximity to each other. In eukaryotic cells, things are entirely different. Prior to becoming TFs, the protein molecules have to travel from cytoplasm to nucleus by penetrating the nuclear membrane and then finding the sequence-specific regulatory site. The mechanism of how exactly this happens is a major and largely unresolved mystery in biophysics. In a large body of the literature on the topic, three sources^54^–^56^ are especially helpful in elucidating physical aspects of protein translocation and in providing comprehensive reviews. This question has been also recently visited in the work by this author.^57^ Within the context of chemical kinetics, the proteins’ travel time may be accounted for by introducing the set of *delay intervals* for the protein variables. A straightforward way of doing this is replacement of the mRNA production terms $\gamma_{i}\prod_{k=1}^{N}{\left[p_{k}(t)\right]}^{P_{i\;k}}\text{by}\;\gamma_{i}\prod_{k=1}^{N}{\left[p_{k}(t-\zeta_{k})\right]}^{P_{i\;k}}$, where {*ζ_k_*} is a set of protein-specific delays. Such a measure would move the problem from the realm of ordinary differential equations to the realm of mathematically much more complex *delay equations*. It is far beyond the scope of this paper to delve deeper into this problem. However, it is possible to envision some general tendencies associated with the introduction of delays. Assuming that the delay times are small as compared to the characteristic times of chemical reactions, we obtain

(1.18) $$\begin{array}{l} \prod_{k=1}^{N}{\left[p_{k}(t-\zeta_{k})\right]}^{P_{i\;k}}\\ \approx \prod_{k=1}^{N}{\left[p_{k}(t)\right]}^{P_{i\;k}}\text{exp}\left\{\sum_{k=1}^{N}P_{ik}\left(\beta_{k}-\alpha_{k}\frac{r_{k}}{p_{k}}\right)\zeta_{k}\right\} \end{array}$$

As seen from (1.18), the problem is now transformed into a more complex one, but generally remains in the same class of nonlinear problems as in the well stirred systems. A new feature is that the mRNA production term has become dependent on the presence of other mRNAs. Obviously, such a complication is not conducive to more stability because it introduces additional feedbacks into the process of gene expression and additional uncertainty associated with random kinetic rates.

## 7. Qualitative Picture of Instability

The mathematical description of instability offered above is a formal representation of a wide class of phenomena frequently observed in networks. Among them are traffic jams, stampedes, instabilities of combat operations, fluctuations in predatorprey populations, blackouts of power grids, stock market panics, and others.^58^ Suppose that at a certain moment in time all the protein supply lines have been perfectly balanced with all the mRNA transcription rates, thus rendering equilibrium to the regulatory system. Suppose also that due to some random events comprising a rich picture of intracellular stochasticity (abundantly discussed in the literature, see^43^,^59^ and references therein), a certain protein, say *p_awol_*, failed to report to the designated regulatory site. Obviously, transcription activity of the corresponding gene (say, gene-X) will be temporarily halted, and the mRNA-X molecule will not be synthesized. This also means that the protein translated from the mRNA-X, let’s call it protein-Y, a much needed TF for regulation of the gene-Y, will also fail to appear at the scene. These failures will propagate further to other genes. Quite accidentally, it may also happen that on a parallel sequence of events, an mRNA-Z is destroyed prior to reaching a ribosome, thus creating a deficit of the protein-Z followed by its own domino effect of the regulatory failures. It is easy to envision that, since there are 25,000 genes in the genome and each requires from 30 to 100 TFs, such unfortunate coincidences should be quite frequent. Due to massive high order interactions, perturbations that appeared somewhere in the system easily penetrate to other domains of regulation creating unidirectional progression to deregulation. It is obvious that in such an environment, nothing like an orderly protein assembly line may exist. Spontaneous failures like traffic jams, bottlenecks, backlogs, delays, loss of synchronization, etc., are unavoidable circumstances surrounding their functioning. Each successful transcription in a dynamically unstable system may be only thought of as a comparatively rare and sporadic event. In^43^ such events have been termed as instances of *stochastic cooperativity*.

A number of observations support the view of transcription as a sequence of sporadic events (see more detailed discussions in^44^,^57^.) Recent experiments^60^ demonstrated that even in an individual cell, the production of a protein and supporting enzymes is a stochastic process following a complex pattern of *bursting* with random distribution of intensities and durations. Similarly, it was found in^61^ that quantitative relations between transcription factor concentrations and the rate of protein production “fluctuate dramatically in individual living cells, thereby limiting the accuracy with which genetic transcription circuits can transfer signals.” The phenomenon of burstiness is wide spread in genetic regulation. Thus, the authors of^62^ report that “transcription occurs in pulses in muscle fibers.” In Ref.^63^, it was found that “transcription of individual genes in eukaryotic cells occurs randomly and infrequently.” Similar observations have been made in.^64^–^67^

Is there any alternative to sporadicity of genetic regulation? A common counterargument is that *massive redundancy* is a recipe against instabilities and sporadicity. In the context of gene expression, the redundancy could mean that the proteins of each type serving as TFs somewhere throughout the genome are always available in the vicinity of any regulatory site (let’s call this type of redundancy *copy number redundancy*.) However, it should be taken into consideration that the vast majority of TFs in a cell are produced within the same cell, and the gene expression machinery is their only source. Therefore, it is a more or less closed supply-demand production cycle, without many leftovers or excesses. The hypothesis of copy number redundancy would require availability of many more proteins than the gene expression network is capable of synthesizing. In a sense, it may be said that there is always a shortage of regulatory proteins, and their availability depends on the efficiency of the gene expression production line itself.

It is also conceivable that yet another type of redundancy, a *functional redundancy*, may exist. In the context of gene expression, functional redundancy would mean that there are many ways to synthesize the same protein thus providing alternative modes of functioning in spite of sporadicity and instabilities. It should be noted, however, that functional redundancy, if it exists, would not be a way of eliminating or suppressing instabilities; rather, it would be the way of resilience in the face of and coexistence with instabilities.

## 8. Interpretation of the Jacobian Eigenvalue Spectra

Given some initial conditions within a small ɛ-vicinity of the fixed point, ${\text{x}}_{\varepsilon }^{0}=\{{\text{p}}_{\varepsilon }^{0},{\text{r}}_{\varepsilon }^{0}\}$, the subsequent evolution of the system is described by the equation

(1.19) $$\begin{array}{ll} \text{x}(t) & =\text{exp}(\text{J}t){\text{x}}_{0}\\ & =\{{\text{Z}}^{-1}\text{diag}[\text{exp}(\lambda_{1}t),\ldots ,\text{exp}(\lambda_{N}t)]\text{Z}\}{\text{x}}_{\varepsilon }^{0} \end{array}$$

(e.g.^68^ Chapter 3), where **Z** is the similarity matrix (in this case, simply having the eigenvectors of **J** as its columns.) All the eigenvalues may be ordered as follows

(1.20) $$\lambda_{\text{min}},\{\lambda_{\text{min}}<\text{Re}\lambda <0\},0,\{0<\text{Re}\lambda <\lambda_{\text{max}}\},\lambda_{\text{max}}$$

The first group containing just one real negative eigenvalue, λ_min_, describes rapid attenuation of the perturbation, $\left|{\text{x}}_{\varepsilon }^{0}-{\text{x}}^{0}\right|$, and relaxation toward the equilibrium point, **x**^0^. The second group, {λ_min_ < Re λ < 0}, describes oscillatory decay of the perturbations with a variety of attenuation times and periods. All the eigenvalues in this group comprise the *stable manifold*. The third group, Re λ = 0, called the *center manifold*, contains only stationary periodic oscillations (unless Im λ = 0.) The group {0 < Re λ < λ_max_}, the *unstable manifold*, describes oscillatory growth of disturbances and moving the system further from the equilibrium. Finally, the last member in (1.20), λ_max_, describes rapid, purely exponential growth of the disturbances. As seen from examples Figures 1–5, typically the largest eigenvalue, λ_max_, is substantially greater than the moduli of the rest of the eigenvalues. This means that all the numerous transitory details of behavior manifested in the spectra are comparatively unimportant as they become quickly overridden by the rapid exponential growth of just one term, exp(λ_max_ *t*). It also means that the initial state, **x**^0^, which is generally representable as a linear combination of all eigenvectors, **x**^0^ = *c*_1_ **e**_λ_1__ +*c*_2_ **e**_λ_2__ +... +*c*_N_**e**_λ_max__, without loss of generality, may be reduced to the last term, *c_N_***e**_λ_max__. As shown above, the largest eigenvalue is dependent only on the complexity, λ_max_ = ζ^½^ – 1. We come, therefore, to an important conclusion that the characteristic time of existence of the equilibrium state rapidly decreases with increasing complexity. It is also worth mentioning that kinetic rates have a negligible impact on this conclusion; the time of decay is overwhelmingly dependent on the system’s topology, that is, on its link density.

## 9. Long Term Behavior

Jacobian analysis of stability in the vicinity of the fixed point (often called *linear stability*) provides little guidance regarding the patterns of long term behavior of the system. Lessons learned from the studies of low dimensional nonlinear systems show that their behavior may be extremely complex, often manifesting the patterns of deterministic chaos, as vividly demonstrated by classical examples of Lorenz attractor and Lotka-Volterra population dynamics.^49^ Intuition tells us that there is little hope that in highly nonlinear systems of very large dimension—and genetic regulatory networks are such systems—the behavior may miraculously become more orderly. Recently, an interesting argument was put forward by Dechert et al.^69^ Using neural network as a model for the system’s dynamics, he came to the conclusion that as the dimension and the complexity of the network increase, the probability of chaotic behavior increases to 100%. Since neural networks are *dense* in the set of dynamical systems, the authors conjecture that most large dynamical systems ought to be chaotic. Such a conclusion is in agreement with an earlier argument by Brock that “the larger the dimension of a nonlinear dynamical system, the larger the probability that the system dynamics have a positive Lyapunov exponent.”^70^

As mentioned in Section 3, the scenario of asymptotic stability assumes that, starting from some arbitrary initial conditions within the basin of attraction, all the trajectories of a dynamical system converge to a certain domain of the phase space and stay within it forever. However, the behavior of trajectories within that domain may be quite different. It may assume the form of orderly quasi-periodic motion (in this case, it is called *limit cycle*), but it also may become chaotic with exponentially fast increasing of distances between initially close trajectories. Two questions are of key importance for understanding the long-term behavior. First, one needs to establish the sign of the *phase space compressibility*. Negative compressibility means that the volume occupied by a bunch of trajectories decreases with time; that would generally indicate asymptotic stability.^71^ However, existence of asymptotic stability does not preclude the attractor being chaotic; the Lyapunov Exponent analysis is the tool capable of resolving this issue.^72^ It is generally accepted that if the Lyapunov spectrum contains at least one positive exponent then the system is chaotic. Geometrically, this means that ever decreasing phase space volume nevertheless becomes folded in progressively thinner layers, deformed in numerous branches and tentacles with a tendency to reach even the farthest domains of the phase space.

Phase space compressibility for the system (1.4) is easy to calculate. For this purpose we use the following form of the equations of chemical kinetics

(1.21) $$\frac{dx_{i}}{dt}=f_{i}(\text{x})=\frac{1}{\tau_{i}}\left\{\text{exp}\left[\sum_{k=1}P_{ik}^{I}x_{k}\right]-\text{exp}\left[\sum_{k=1}Q_{ik}^{I}x_{k}\right]\right\},$$

where $P_{ik}^{I}=P_{ik}-\delta_{ik}$ and $Q_{ik}^{I}=Q_{ik}-\delta_{ik}$. The Jacobian matrix at an arbitrary point, **x**, is

(1.22) $$\begin{array}{ll} J_{ij} & =\frac{\partial f_{i}(\text{x})}{\partial x_{j}}\\ & =\frac{1}{\tau_{i}}\left[P_{ij}^{I}\text{exp}\left(\sum_{k=1}^{N}P_{ik}^{I}x_{k}\right)-Q_{ij}^{I}\text{exp}\left(\sum_{k=1}^{N}Q_{ik}^{I}x_{k}\right)\right] \end{array}$$

Suppose that a constituent, *x_i_*, is not *autocatalytic.* In the context of gene expression, this means that among the TFs for the gene “*i*”, there are no proteins translated from the same gene. In this case, $P_{ii}^{I}=-1$. Further, we note that according to (1.4) all the degradation terms are linear in the corresponding constituents; therefore, $Q_{ii}^{I}=0$. By definition, the *phase space compressibility* is the divergence of the vector field, **f**. Thus,

(1.23) $$\sum_{i=1}\frac{\partial f_{i}(\text{x})}{\partial x_{i}}=\sum_{i=1}J_{ii}=-\sum_{i=1}\frac{1}{\tau_{i}}\text{exp}\left(\sum_{k=1}P_{ik}^{I}x_{k}\right)$$

In this scenario, the phase space volume is contracting; this is an indication of asymptotic stability.

It is of interest to examine a more complex case when at least some gene expressions are autocatalytic but require participation of only one protein expressed by the same gene. It is easy to see from general expressions (1.23) and (1.24) that this assumption does not change the sign of phase space compressibility since the corresponding terms simply vanish. In an even more complex, but less likely, scenario when the autocatalytic self-reproduction requires participation of more than one protein expressed from the same gene, the sign of phase space compressibility may vary and becomes dependent on many circumstances. It is quite possible that in some of these cases, the system will lose the property of asymptotic stability because high-order self-reproduction is equivalent to the existence of a strong positive feedback loop. It is also worth mentioning that all the scenarios of *self-destruction* (i.e. the degradations supported by the same constituents as those being degraded) cause $Q_{ii}^{I}>0$, thus leaving the sign of phase space compressibility unchanged and even increasing its absolute value.

Computation of Lyapunov exponents is a daunting task even for comparatively simple systems,^73^ and it seems to be a hopeless undertaking to apply standard techniques^72^ to a system with dimensions in the thousands. However, high dimensionality may be of some advantage and serve as a basis for approximations and simplifications.^43^ Detailed structure of the asymptotic regime, whether it is a chaotic attractor or a limit cycle, is not of primary importance for understanding dynamics in systems of very high dimensions. For practical purposes, it is reasonably sufficient just to know that the phase space compressibility is negative, therefore some kind of asymptotic regime does exist. According to general principles of statistical mechanics, rapidly fluctuating variables can always be considered stochastically and treated as random noise disturbing slower evolution of the system. Such an approach automatically leads to the replacement of deterministic differential equations (1.4) by stochastic differential equations and transition to description in probabilistic terms using the Fokker-Plank equation.^74^

## 10. Discussion

When theorists and computational scientists try to capture the transcription-translation mechanism in the language of differential equations, importance of the mRNA decay rates immediately jumps into focus. It is not the goal of this paper to claim that this importance is poorly understood. The point is that in the majority of existing routine microarray protocols, the mRNA decay rates are not measured, remain unknown and are not taken into consideration when making the inferences regarding the states of corresponding genes. There is nobody to blame here. Measurements of the decay rates require special, and in fact very complex, laboratory settings; at this time they are mostly of experimental sort rather than a routine, simple and commonly available component of expression profiling.

The question raised in this paper is not simply a question of *poor reliability* of microarray measurements. The question is deeper. We are making the point that measurements of mRNA abundances are not *fully representative* of gene activity. It often happens in many areas of science that direct measurements of the quantities of interest are not possible, but instead some proxies or surrogate variables can be observed. A textbook example is that we cannot directly measure the temperature of stars. What can be observed and measured are only the spectra of their electromagnetic radiation. But prior to inferring the temperature of a star from the analysis of its radiation, a theory should exist that relates the temperature to the radiation spectrum. Inferring the quantity of interest (temperature) from the quantities observed (spectrum) constitutes the solution of an *inverse* problem. It is quite a common situation that inverse problems are either ill-conditioned or not solvable at all. This is because solution of the inverse problem would require a number of additional quantities, which in turn require their own independent measurements and even more comprehensive theories. The situation with the relations between transcription levels and transcription rates is quite similar: transcription rates are the *unobservable* quantities of interest, whereas transcription levels are the quantities being actually observed. It is true that transcription rates directly impact the transcription levels. But it is also true that many other processes may impact transcription levels. This is why the inverse problem of derivation of transcription rates from the transcription levels is ill-conditioned and may be not solvable.

The consequences of simplified thinking and lack of logically self-consistent view on the nature of transcription profiling may have detrimental effect, especially in clinical settings. Relative simplicity of transcription profiling with microarrays makes them an attractive tool for clinical diagnostics. A medical practitioner would not hesitate to jump directly from the observed abnormal mRNA abundances to conclusion regarding abnormality of corresponding genes. The author is trying to convey the idea that there is no such direct link and cannot be due to fundamental reasons lying in the very basis of intracellular biochemistry.

Observed biological robustness of living organisms is not a counterargument to dynamical biochemical instability. *Robustness* differs from *stability* in that it deals with maintaining *the system’s functions* as opposed to *the system’s states*.^75^ The seeming contradiction between functional stability of a vast organizational structure consisting of a large number of biochemical networks and possible dynamical instability in each of them is fictitious; it attempts to oppose different levels of biological organization. A logically satisfactory way of looking into these issues is through the paradigm called *dual causality* formulated by Palsson.^76^ He writes: “Unlike physiochemical sciences, biology is subject to dual causality or dual causation. Biology is governed not only by the natural laws but also by genetic programs. Thus, while biological functions obey the natural laws, their functions are not predictable by the natural laws alone. Biological systems function and evolve under the confines of the natural laws according to basic biological principles, such as generation of diversity and natural selection. The natural laws can be described based on physicochemical principles and used to define the constrains under which organisms must operate. How organisms operate under these constrains is a function of their evolutionary history and survival.” Within this paradigm of *dual causality*, inherent dynamical instability represents the “natural laws” and “physicochemical principles” whereas biological robustness is a result of evolutionary history in which this dynamical instability is effectively used for gaining evolutionary advantages and survival.

## 11. Summary

A nonlinear dynamical model for the description of gene expression has been introduced. The model is based on the equations of chemical kinetics and explicitly takes into consideration nonlinear gene-to-gene interaction through the teamwork of proteins serving as transcription factors.

It has been shown that there is a unique fixed point in this model; Jacobian analysis of stability has been performed for this fixed point. Numeric computation of the eigenvalue spectra revealed a high degree of dynamical instability inherent in such systems. The core element responsible for the emergence of instability is nonlinearity of the equations of chemical kinetics associated with team-work of proteins associated with transcription. Instability occurs every time when the average number of proteins (per gene) serving as transcription factors becomes greater than one. With the typical number of transcription factors from 30 to 100 per gene, genetic regulatory systems lie deeply within the domain of dynamical instability.

It has been demonstrated analytically, and confirmed numerically, that a simple rule exists connecting the network’s link density with the degree of its instability. Namely, the largest eigenvalue of the Jacobian spectrum, λ_max_, the key quantity characterizing instability, is directly related to the *complexity* of the system measured by the network’s *link density*, ζ: λ_max_ = ζ^½^ – 1. It has been shown that this relation is highly robust and holds under widely varying assumptions regarding probabilistic distributions of kinetic rates and stoichiometric coefficients. It may be claimed with a high degree of certainty that the network’s instability is mainly determined by its link density and largely independent of its kinetics.

It has been shown by direct analytical calculation that long-term behavior of the regulatory system, under some natural assumption of absence of self-reproducibility, represents a dissipative flow with negative phase space compressibility. This guarantees asymptotic stability of the system and existence of some sort of attractor. The question whether or not this attractor is chaotic or, alternatively, represents a multispectral quasi-periodic limit cycle remains open at this time. This question, however, is of secondary importance in systems with the dimensions in the thousands.

Direct relations between transcription levels and transcription rates are only possible in the state of lasting steady state equilibrium. But even in such a hypothetical state, inference of transcription rates from transcription levels is not possible unless an additional set of quantities is measured in the same experimental settings; these quantities are the rates of mRNA degradation.

The aforementioned result that the largest Jacobian eigenvalue, λ_max_, bears a simple relationship to the network link density, ζ, that is, λ_max_ = ζ^½^ – 1, may be of interest beyond the scope of this paper and useful in a wider context of network dynamics.

## 12. Conclusion

A key assumption underlying microarray measurements is that measuring mRNA abundances provides the basis for definitive statements regarding the functionality and integrity of the corresponding genes. In particular, excess or deficiency in mRNA copy numbers is considered to be an indicator of possible damage to their genetic codes. In formal terms, such reasoning assumes the existence of a direct link between transcription levels and transcription rates. As shown above, such a direct link is only possible in the state of stable equilibrium. Inherent dynamical instability and sporadicity of genetic regulation elucidated in this paper make such a direct link highly questionable. There may be many reasons other then genetic mutation for the mRNA abundances being up-or down-regulated.

There is no doubt that the analysis of the transcriptome, along with the proteome and the metabolome, may serve as a valuable diagnostic tool. However, the logical leap from the state of transcriptome to the state of genome is not self-evident and requires much more careful substantiation than is currently accepted in experimental biology. Better understanding of the essence of microarray measurement may help in developing more efficient protocols for use in clinical practice. Alternatively, it is conceivable that such an in-depth analysis may lead to realization that a tool that is inherently unstable is not expected to be a reliable instrument for patient-related decision making.

## References

[1] Schena M Parallel human genome analysis: microarray-based expression monitoring of 1000 genes Proc Natl Acad Sci U S A 1996 93 10614 8855227 10.1073/pnas.93.20.10614 PMC38202

[2] Schena M Shalon D Davis RW Brown PO Quantitative monitoring of gene expression patterns with a complementary DNA microarray Science 1995 270 467 7569999 10.1126/science.270.5235.467

[3] Shalon D Smith SJ Brown PO A DNA microarray system for analyzing complex DNA samples using two-color fluorescent probe hybridization Genome Res 1996 6 639 8796352 10.1101/gr.6.7.639

[4] DeRisi J Use of a cDNA microarray to analyse gene expression patterns in human cancer Nat Genet 1996 14 457 8944026 10.1038/ng1296-457

[5] Lander ES Array of hope Nat Genet 1999 21 3 9915492 10.1038/4427

[6] Brown PO Botstein D Exploring the new world of the genome with DNA microarrays Nat Genet 1999 21 33 9915498 10.1038/4462

[7] Duggan DJ Bittner M Chen Y Meltzer P Trent JM Expression profiling using cDNA microarrays Nat Genet 1999 21 10 9915494 10.1038/4434

[8] Ioannidis JP Microarrays and molecular research: noise discovery? Lancet 2005 365 454 15705441 10.1016/S0140-6736(05)17878-7

[9] Ioannidis JP Is molecular profiling ready for use in clinical decision making Oncologist 2007 12 301 17405894 10.1634/theoncologist.12-3-301

[10] Shi L The MicroArray Quality Control (MAQC) project shows inter-and intraplatform reproducibility of gene expression measurements Nat Biotechnol 2006 24 1151 16964229 10.1038/nbt1239 PMC3272078

[11] Kadonaga JT Regulation of RNA polymerase II transcription by sequence-specific DNA binding factors Cell 2004 116 247 14744435 10.1016/s0092-8674(03)01078-x

[12] Baldi P Long AD A Bayesian framework for the analysis of microarray expression data: regularized t -test and statistical inferences of gene changes Bioinformatics 2001 17 509 11395427 10.1093/bioinformatics/17.6.509

[13] Mestl T Plahte E Omholt SW A mathematical framework for describing and analysing gene regulatory networks J Theor Biol 1995 176 291 7475117 10.1006/jtbi.1995.0199

[14] Sebastiani P Yu YH Ramoni MF Bayesian machine learning and its potential applications to the genomic study of oral oncology Adv Dent Res 2003 17 104 15126219 10.1177/154407370301700124

[15] Ramoni MF Sebastiani P Kohane IS Cluster analysis of gene expression dynamics Proc Natl Acad Sci U S A 2002 99 9121 12082179 10.1073/pnas.132656399 PMC123104

[16] Gardner TS di BD Lorenz D Collins JJ Inferring genetic networks and identifying compound mode of action via expression profiling Science 2003 301 102 12843395 10.1126/science.1081900

[17] Yu J Smith VA Wang PP Hartemink AJ Jarvis ED Advances to Bayesian network inference for generating causal networks from observational biological data Bioinformatics 2004 20 3594 15284094 10.1093/bioinformatics/bth448

[18] Margolin AA ARACNE: an algorithm for the reconstruction of gene regulatory networks in a mammalian cellular context BMC Bioinformatics 2006 7 Suppl 1 S7 16723010 10.1186/1471-2105-7-S1-S7 PMC1810318

[19] Toyoshiba H Gene interaction network suggests dioxin induces a significant linkage between aryl hydrocarbon receptor and retinoic acid receptor beta Environ Health Perspect 2004 112 1217 15345368 10.1289/txg.7020 PMC1277115

[20] Thomas R Paredes CJ Mehrotra S Hatzimanikatis V Papoutsakis ET A model-based optimization framework for the inference of regulatory interactions using time-course DNA microarray expression data BMC Bioinformatics 2007 8 228 17603872 10.1186/1471-2105-8-228 PMC1940027

[21] Chen T He HL Church GM Modeling gene expression with differential equations Pac Symp Biocomput 1999 29 10380183

[22] deJong H Modeling and simulation of genetic regulatory systems: a literature review J Comput Biol 2002 9 67 11911796 10.1089/10665270252833208

[23] Voit EO Radivoyevitch T Biochemical systems analysis of genome-wide expression data Bioinformatics 2000 16 1023 11159314 10.1093/bioinformatics/16.11.1023

[24] Gibson M Mjolsness E Modeling the activity of single genes Bower JM Bolouri H MIT Press Cambridge, MA 2001

[25] Bernstein JA Khodursky AB Lin PH Lin-Chao S Cohen SN Global analysis of mRNA decay and abundance in Escherichia coli at single-gene resolution using two-color fluorescent DNA microarrays Proc Natl Acad Sci U S A 2002 99 9697 12119387 10.1073/pnas.112318199 PMC124983

[26] Garcia-Martinez J Aranda A Perez-Ortin JE Genomic run-on evaluates transcription rates for all yeast genes and identifies gene regulatory mechanisms Mol Cell 2004 15 303 15260981 10.1016/j.molcel.2004.06.004

[27] Cheadle C Stability regulation of mRNA and the control of gene expression Ann N Y Acad Sci 2005 1058 196 16394137 10.1196/annals.1359.026

[28] Cheadle C Control of gene expression during T cell activation: alternate regulation of mRNA transcription and mRNA stability BMC Genomics 2005 6 75 15907206 10.1186/1471-2164-6-75 PMC1156890

[29] Rosenfeld S Stochastic Oscillations in Genetic Regulatory Networks EURASIP Journal of Bioinformatics and Systems Biology 2006 1 10.1155/BSB/2006/59526 PMC3171319 18427584

[30] Rhodius VA LaRossa RA Uses and pitfalls of microarrays for studying transcriptional regulation Curr Opin Microbiol 2003 6 114 12732299 10.1016/s1369-5274(03)00034-1

[31] Savageau MA Biochemical systems analysis. 3. Dynamic solutions using a power-law approximation J Theor Biol 1970 26 215 5434343 10.1016/s0022-5193(70)80013-3

[32] Voit EO S-System Approach to Understanding Complexity Van Norstand Reinhold NY Canonical Nonlinear Modeling 1991

[33] Savageau MA Biochemical systems analysis. I. Some mathematical properties of the rate law for the component enzymatic reactions J Theor Biol 1969 25 365 5387046 10.1016/s0022-5193(69)80026-3

[34] Savageau MA Biochemical systems analysis. II. The steady-state solutions for an n-pool system using a power-law approximation J Theor Biol 1969 25 370 5387047 10.1016/s0022-5193(69)80027-5

[35] Voit EO Savageau MA Accuracy of alternative representations for integrated biochemical systems Biochemistry 1987 26 6869 3427048 10.1021/bi00395a042

[36] Voit EO Computational Analysis of Biochemical Systems: A Practical Guide for Biochemists and Molecular Biologists Cambridge University Press Cambridge, UK 2000

[37] Thomas R Mehrotra S Papoutsakis ET Hatzimanikatis V A model-based optimization framework for the inference on gene regulatory networks from DNA array data Bioinformatics 2004 20 3221 15247105 10.1093/bioinformatics/bth389

[38] Kirkilionis M Reaction systems, graph theory and dynamical networks Gauges R Kummer U Pahle J Willy P Fifth Workshop on Computation of Biochemical Pathways and Genetic Networks University of Heidelberg Heidelberg 2008

[39] Rosenfeld S Origins of Stochasticity and Burstiness in High-Dimensional Biochemical Networks EURASIP Journal of Bioinformatics and Systems Biology 2008 10.1155/2009/362309 PMC3171425 18946549

[40] Rosenfeld S Why do high-dimensional networks seem to be stable? A reflection on stochasticity of dynamically unstable nonlinear systems Gauges R Kummer U Pahle J Willy P Fifth Workshop on Computation of Biochemical Pathways and Genetic Networks University of Heidelberg Heidelberg 2008 101 12

[41] Rosenfeld S Mathematical Description of Biochemical Networks: instability, stochasticity, evolution Thiangliam S Systems Biology of Cancer Oxford University Press 2009

[42] Rosenfeld S Kapetanovic I Systems Biology and Cancer Prevention: All Options on the Table Gene Regulation and Systems Biology 2008 2 307 19787092 10.4137/grsb.s1114 PMC2733099

[43] Rosenfeld S Stochastic cooperativity in non-linear dynamics of genetic regulatory networks Math Biosci 2007 210 121 17617426 10.1016/j.mbs.2007.05.006

[44] Rosenfeld S Origins of Stochasticity and Burstiness in High-Dimensional Biochemical Networks EURASIP Journal of Bioinformatics and Systems Biology 2009 10.1155/2009/362309 PMC3171425 18946549

[45] Chin CF Shih AC Fan KC Influence of mRNA decay rates on the computational prediction of transcription rate profiles from gene expression profiles J Biosci 2007 32 1251 18202449 10.1007/s12038-007-0134-9

[46] Wang W Cherry JM Botstein D Li H A systematic approach to reconstructing transcription networks in Saccharomyces Cerevisiae Proc Natl Acad Sci U S A 2002 99 16893 12482955 10.1073/pnas.252638199 PMC139240

[47] Sharova LV Database for mRNA half-life of 19 977 genes obtained by DNA microarray analysis of pluripotent and differentiating mouse embryonic stem cells DNA Res 2009 16 45 19001483 10.1093/dnares/dsn030 PMC2644350

[48] Hambraeus G von WC Hederstedt L Genome-wide survey of mRNA half-lives in Bacillus subtilis identifies extremely stable mRNAs Mol Genet Genomics 2003 269 706 12884008 10.1007/s00438-003-0883-6

[49] Guckenheimer J Holmes P Nonlinear Oscillations, Dynamical Systems, and Bifurcations of Vector Fields Springer 2002

[50] Gantmacher FR Applications of the Theory of Matrices Interscience NY 1959

[51] Kirkilionis M Reaction systems, graph theory and dynamical networks Gauges R Kummer U Pahle J Willy P University of Heidelberg Heidelberg 2008

[52] Bailey JE Complex biology with no parameters Nat Biotechnol 2001 19 503 11385433 10.1038/89204

[53] Tournier L Approximation of dynamical systems using S-Systems theory: Application to biological systems International Symposium on Symbolic and Algebraic Computations 2005 317 24

[54] Slutsky M Protein-DNA Interaction, Random Walk and Polymer Statistics, PhD thesis, MIT, Physics Dpt.2005

[55] Coppey M Benichou O Voituriez R Moreau M Kinetics of target site localization of a protein on DNA: a stochastic approach Biophys J 2004 87 1640 15345543 10.1529/biophysj.104.045773 PMC1304569

[56] Halford SE Szczelkun MD How to get from A to B: strategies for analysing protein motion on DNA Eur Biophys J 2002 31 257 12122472 10.1007/s00249-002-0224-4

[57] Rosenfeld S Mathematical Description of Biochemical Networks: instability, stochasticity, evolution Thiangliam S Systems Biology of Cancer Oxford University Press 2009

[58] Ball R Holmes P Dynamical systems, stability, and chaos Denier JP Frederiksen JS Frontiers in Turbulence and Coherent Structures World Scientific Singapore 2007 1 27

[59] Rosenfeld S Stochastic Oscillations in Genetic Regulatory Networks. Applications to Microarray Experiment Rizzi A Vichi M COMPSTAT-2006 Physica-Verlag 2006 1609 18 10.1155/BSB/2006/59526 PMC3171319 18427584

[60] Cai L Friedman N Xie XS Stochastic protein expression in individual cells at the single molecule level Nature 2006 440 358 16541077 10.1038/nature04599

[61] Rosenfeld N Young JW Alon U Swain PS Elowitz MB Gene regulation at the single-cell level Science 2005 307 1962 15790856 10.1126/science.1106914

[62] Newlands S Transcription occurs in pulses in muscle fibers Genes Dev 1998 12 2748 9732272 10.1101/gad.12.17.2748 PMC317123

[63] Ross IL Browne CM Hume DA Transcription of individual genes in eukaryotic cells occurs randomly and infrequently Immunol Cell Biol 1994 72 177 8200693 10.1038/icb.1994.26

[64] Blake WJ Kaern M Cantor CR Collins JJ Noise in eukaryotic gene expression Nature 2003 422 633 12687005 10.1038/nature01546

[65] Golding I Cox EC RNA dynamics in live Escherichia coli cells Proc Natl Acad Sci U S A 2004 101 11310 15277674 10.1073/pnas.0404443101 PMC509199

[66] Golding I Paulsson J Zawilski SM Cox EC Real-time kinetics of gene activity in individual bacteria Cell 2005 123 1025 16360033 10.1016/j.cell.2005.09.031

[67] Yu J Xiao J Ren X Lao K Xie XS Probing gene expression in live cells, one protein molecule at a time Science 2006 311 1600 16543458 10.1126/science.1119623

[68] Teschl G Ordinary Differential Equations and Dynamical Systems Lecture Notes 2008

[69] Dechert WD Sprott JC Albers DJ On the Probability of Chaos in Large Dynamical Systems Journal of Economic Dynamics and Control 1999 23 1197

[70] Brock WA Pathways to randomness in the economy: emergent nonlinearity and chaos in economics and finance Estudios Economicos 1993 8 3

[71] Strogatz S Nonlinear Dynamics and Chaos: With applications to Physics, Biology, Chemistry and Engineering Springer-Verlag 1994

[72] Greene JM Kim JS The Calculation of the Lyapunov Spectra Physica 24D 1987 213

[73] Geist K Parlitz U Lauterborn W Comparison of Different Methods for Computing Lyapunov Exponents Progress of Theoretical Physics 1990 3 875

[74] Zwanzig R Ensemble method in the theory of reversibility The Journal of Chemical Physics 1960 33 1338

[75] Kitano H Biological robustness Nat Rev Genet 2004 5 826 15520792 10.1038/nrg1471

[76] Palsson B Systems Biology Properties of Reconstructed Networks Cambridge University Press 2006

